# Can AI Generate Useful Messages for Smoking Cessation Campaigns? A Test with Different Emotional Appeals and Source Attribution

**DOI:** 10.3390/ijerph22101540

**Published:** 2025-10-09

**Authors:** Wan-Lun Chang, Xiaomei Cai, Xiaoquan Zhao

**Affiliations:** Department of Communication, George Mason University, Fairfax, VA 22030, USA; wchang23@gmu.edu (W.-L.C.); xcai@gmu.edu (X.C.)

**Keywords:** tobacco education campaign, AI-generated messages, ChatGPT, emotional appeals, source attribution

## Abstract

This study investigates the viability of using ChatGPT 3.5 to produce smoking cessation messages featuring different emotional appeals. The effect of source attribution to Artificial Intelligence (AI) vs. human experts is also examined. A sample of current smokers (*N* = 480) was recruited from Prolific and randomly assigned to read one of five ChatGPT-generated messages reflecting a 2 (appeal: threat vs. humor) × 2 (source: AI vs. human experts) factorial design plus an irrelevant message control condition. Exposure to the smoking cessation messages led to a pattern of cognitive and emotional responses largely consistent with expectations based on previous research. Compared to control, the smoking cessation messages generated greater risk perceptions on the featured health effects but did not produce significantly stronger intentions to quit. Human experts as the source produced greater perceived source credibility than AI, but there was no source effect on other outcomes. No interaction between message appeals and source attribution was observed. Implications of the findings for tobacco education campaigns are discussed.

## 1. Introduction

The development of artificial intelligence (AI) has profoundly transformed human life, including how people communicate. From an application perspective, AI communication research has primarily focused on AI-assisted one-on-one interactions, the persuasive power of AI-generated messages, comparisons with human-generated messages, and human responses to the disclosure of AI versus human message sources [1,2,3]. However, few studies have examined the persuasive effects of AI-generated emotional appeal messages in the context of broad-reaching health education campaigns. Additionally, audience preferences for AI versus human sources in campaign messaging remain under-researched and are likely to be both issue- and population-dependent.

To address these gaps, the current study investigates the persuasive effects of AI-generated textual messages for potential use in smoking cessation campaigns targeting adults who currently smoke. Despite decades of intervention efforts, tobacco use remains a leading cause of preventable diseases around the globe and is responsible for over 7 million premature deaths annually [4]. Media campaigns have been recognized as an effective method for promoting smoking cessation among individuals who smoke [4,5]. Campaign success requires high-quality messages that are sufficiently large in quantity to sustain exposure among the target audience. However, human-led message development can be labor-intensive and time-consuming, potentially limiting the amount and variety of messages available for campaigns to deploy. In this study, we examine whether AI-generated messages about the health consequences of smoking featuring threat versus humor appeals elicit audience responses consistent with the intended message design. We further examine whether these messages can influence smoking risk perceptions and quitting intentions, both important antecedents to smoking behavior according to the reasoned action approach to behavioral prediction [6]. Finally, we investigate whether source attribution to AI versus human experts results in different cognitive and emotional responses and persuasive outcomes.

### 1.1. AI and Its Application in Campaigns

The emergence and evolution of AI have changed how people communicate, introducing new possibilities, challenges, and complexities [7,8,9]. In many communication applications, AI is tasked with generating persuasive messages to shape people’s perceptions, attitudes, and behaviors [10]. A natural question to ask, then, is whether persuasive messages generated by AI can be effective. Huang and Wang [10] conducted a meta-analysis comparing the effects of AI and human as communication agents on persuasive outcomes across 121 randomized experimental studies (*N* = 53,977). They found that AI and human agents did not differ in their ability to influence perceptions, attitudes, and actual behaviors, but that AI was less effective at shaping behavioral intentions. Interestingly, AI showed a greater impact on actual behaviors than humans in one-way communication (such as AI newscasts) but not in two-way communication (such as live chats or interactive messaging). Based on these findings, the researchers recommended further research in specific application contexts to deepen understanding of AI’s persuasive power.

Much existing research has focused on one-on-one interactions between humans and AI agents. However, some studies have also explored the possibilities of using AI to generate messages for large-scale campaigns. In contrast to one-on-one interactions, campaigns typically rely on broad-reaching media to disseminate informational and persuasive messages to large audiences. While one-on-one messages, such as live chats, may be highly personalized, campaign messages tend to address shared concerns and broad characteristics within the target population. With the assistance of AI, especially large language models, campaign organizations can generate potentially high-quality campaign messages on any given topic efficiently [11,12]. Karinshak et al. [13] provided GPT-3 with prompts that varied in format, length, and examples to generate COVID-19 pro-vaccination messages. These messages were evaluated on content accuracy, relevance, clarity of persuasive intent, edits required, and completeness in information coverage. Although the criteria were not consistently met, findings from the study demonstrated that, overall, AI is capable of generating viable campaign messages when given clear instructions.

In recent years, researchers have started to examine AI’s ability to generate specific types of messages featuring well-defined content and structural characteristics for campaign purposes. For example, Chu and Liu [2] used ChatGPT to generate narratives and non-narratives in both short and long formats about obesity and skin protection. Lim and Schmälzle [12,14] employed Bloom, an open-source multilingual large language model, to generate short awareness-promoting messages suitable for social media use for Folic Acid intake and vaping prevention. These applications provide encouraging evidence that AI may be used to design campaign messages with unique strategic foci and features in different contexts.

### 1.2. AI and Emotions

For their motivating powers, emotions factor prominently in persuasive communication [15,16] and smoking cessation campaigns [17,18,19]. The emotional content of anti-tobacco messages has been recognized as an important contributor to message effectiveness [18,19]. Emotions are an essential component of the human experience, thus also an important focus for AI technologies. The most visible work in this area is AI-generated humor. Humor is a complicated and subjective concept—even humans struggle to fully understand humor. Designing and training AI to generate humor is challenging. According to a study by Avetisyan et al. [20], AI-generated humor can be effective and occasionally novel, though humans typically create humor with better emotional depth and originality. Merritt [21] noted that the limitations of current machine models often require users to repeatedly adjust the prompts to produce good humor. In addition, the effectiveness of AI-generated humor may vary across demographic characteristics, levels of technological proficiency, and contextual and cultural factors [22,23].

In addition to humor, exploring the capabilities of AI to generate other types of emotional messages, as well as people’s recognition and reactions to such messages, is important for the broader use of AI in human communication, in general, and campaign development in specific [24]. Research on AI-generated non-humor appeals is limited. A recent study shows that AI chatbots can react to both positive and negative emotional primes and exhibit distinct response patterns in both risk-taking and prosocial decisions [25]. Such findings suggest the essential capability of emotional reasoning by large language models, supporting the feasibility of using AI to develop different types of emotional messages for persuasion and campaign purposes.

### 1.3. AI Versus Human Sources

Researchers have employed several strategies to compare how people react differently to humans and AI in persuasion. Some experiments present participants with messages generated by either AI or humans, without source disclosure, to compare the effects of the messages themselves [12,13]. Other studies use similar designs, but with source disclosure, to test the influence of accurate source information on audience response [1,13,26]. In yet other studies, the same messages, regardless of actual source, are labeled as either AI- or human-generated to investigate people’s potential bias (either positive or negative) toward different sources [27].

It seems that when the message source is not disclosed, AI-generated messages tend to be well-received, but when the message source is disclosed, people prefer human-generated messages more than AI-generated messages. For example, Karinshak et al. [13] found that when the sources were not disclosed, perceived message quality and persuasiveness of AI-generated pro-vaccination messages were rated higher than real messages used by the CDC in its promotion efforts. When the sources were identified, however, people preferred the CDC messages and reported lower trust in AI-generated vaccination information.

Research has also found situations in which people may prefer AI over humans as the message source. Longoni and Cian [28] demonstrated a “word-of-machine” effect, where people see AI as more competent for utilitarian decisions but prefer human recommendations in hedonic contexts. Dai et al. [26] also suggested that individuals’ evaluations of and reactions to AI communicators may vary significantly across contexts and cultures. These variations highlight the need for further research into how persuasive messages created by and/or attributed to AI sources are received in specific domains of application.

### 1.4. Current Study

This study has several objectives. First, it aims to evaluate the feasibility of using AI to generate relatively elaborate messages for smoking cessation campaigns targeting adults based on carefully curated scientific information on the health consequences of smoking. In previous research, AI-generated messages have typically been short, concise statements suitable for interactive, chat-based messaging. AI’s ability to develop relatively complex messages for health campaigns remains underexplored. Furthermore, as a message development tool, AI’s performance has been shown to vary by context, and its application in tobacco education campaigns is largely untapped. The study hopes to provide insights into the viability of using AI to systematically and cost-effectively produce smoking cessation messages featuring predetermined message strategies. If the quality of AI-generated messages is sufficient, they can become a valuable asset for campaign development.

Second, this study examines AI’s ability to generate messages with specific emotional appeals. In tobacco education campaigns, threat and humor appeals are two widely used message strategies [17,18,29,30,31]. Research has generally pointed to the greater effectiveness of threat over humor appeals in smoking cessation campaigns [18,19]. But humor has known advantages in generating interest and overcoming resistance among hard-to-engage audiences [32,33,34,35]. The need to diversify messaging also suggests utility for the use of humor in smoking cessation campaigns. A study of antismoking ads on YouTube found that 12% of all smoking cessation messages were humor appeals [29]. In an antismoking message archive maintained by the U.S. Center for Disease Control and Prevention, humor (23%) and fear (15%) emerged as the two most commonly used affective appeals, although this study did not differentiate between cessation and prevention ads [17].

Theories of threat appeals, such as the protection motivation theory [36] and the extended parallel process model [37], suggest that including high-threat information in messages, such as information about serious health consequences, may elicit fear responses, increase risk perceptions, and intensify motivations for self-protection. At the same time, high-threat messages may also induce defensive reactions in the forms of perceived threats to freedom, negative thoughts, and counterarguing, among other things [37,38]. Research on humor appeals, on the other hand, suggests that humor can increase positive emotional responses, mitigate negative thoughts, reduce counterarguing, and improve message receptivity in general [32,33]. We want to understand the extent to which AI-generated threat and humor appeal messages may successfully produce patterns of audience reactions consistent with relevant theories and previous campaign research. We would also like to explore the impact of such messages on risk perceptions and intentions to change tobacco use behaviors. According to the reasoned action approach, beliefs about behavioral consequences and intentions to perform a behavior are both important predictors of future behavioral enactment [6]. For this reason, risk perceptions and behavioral intentions are widely accepted as important persuasive outcomes for tobacco education messaging.

Third, this study explores whether people react differently when AI or human experts are identified as the source of smoking cessation messages. Previous research suggests that people’s preference between AI- and human-generated messages tends to vary based on the context and purpose of persuasion [26,28]. In this study, we couple AI vs. humane expert sources with threat vs. humor appeal messages and investigate the extent to which source identity, either alone or in combination with message appeal type, might influence both perceived source credibility and the persuasive effects of tobacco campaign messages.

To address these research objectives, this study uses AI to generate smoking cessation messages targeting adult smokers, featuring relatively novel health consequences of smoking. Emotional appeal (threat vs. humor) and source attribution (AI vs. human experts) are manipulated in the messages. Since this is a proof-of-concept study—i.e., it seeks to examine the viability of using AI to develop usable tobacco cessation messages, not necessarily messages that would outperform those used in current campaigns—a non-health-related message is included as a control. The research questions of the study are as follows:

RQ1: How will cognitive and emotional responses to the smoking cessation message differ from those to the control message?

RQ2: Among the smoking cessation messages, will cognitive and emotional responses, as well as perceived source credibility, vary based on message appeal and source attribution?

RQ3: Will the smoking cessation messages result in greater perceived risk with respect to the message-targeted health effects and greater intentions to quit compared to the control message?

RQ4: Among the smoking cessation messages, will risk perceptions and intentions to quit vary based on message appeal and source attribution?

## 2. Materials and Methods

### 2.1. Design

An online experiment was conducted using a 2 (appeal: threat vs. humor) × 2 (source: AI vs. human experts) factorial design plus an irrelevant message control condition.

### 2.2. Sample

Participants were recruited from Prolific, an online research platform that provides access to a large and diverse panel of participants worldwide. Eligibility criteria included residence in the United States, being 18 and older, currently smoking at least 5 cigarettes per day, and an approval rate of 95–100% in past research participation. Participants received compensation in line with the appropriate hourly wage recommended by Prolific.

### 2.3. Procedure

The study was programmed on Qualtrics and administered through Prolific. The study was described as a health information study without referring specifically to tobacco or cigarette smoking. Participants were screened into the study using existing panel characteristic data already gathered by Prolific. Upon entering the study, participants first indicated informed consent. They then answered the same smoking behavior question as used by Prolific to confirm their eligibility. After that, participants completed additional questions about smoking frequency, dependence, current interest in quitting, and demographic backgrounds. Next, participants were randomized to one of the five experimental conditions (2 × 2 plus control). In each condition except control, participants were presented with one message randomly selected from a set of three that featured the proper manipulation—see Section 2.4. Those in the control condition all saw the same message. Participants were instructed to read the message carefully. After reading, participants first completed a thought-listing procedure and then answered questions on their cognitive and emotional responses to the message. Next, participants indicated the likelihood that they would experience various health problems related to cigarette smoking and intentions to quit. Finally, participants were asked to evaluate the credibility of the message source. The study took an average of 14 min to complete and was approved by the institutional review board at the authors’ home university.

### 2.4. Messages

All messages used in this study were created by ChatGPT 3.5 in 2023. The content of the smoking cessation messages was based on a blog post from the American Lung Association’s website (“10 Health Effects of Smoking You Didn’t Know About”). The blog post presented a list of lesser-known health effects of smoking, drawing from the 2014 Surgeon General’s Report on the health consequences of smoking [39]. The effects were described briefly in the blog post, each with only one or two sentences. Five effects from the list were chosen for message creation in this study, including going blind, erectile dysfunction, hip fractures, rheumatoid arthritis, and gum disease. ChatGPT was prompted to create a 500-word message focusing on these effects using either a ‘serious and somber’ tone or a ‘humorous’ tone. For each message type, three alternative versions were created, enabling random selection of one message for each participant. In the study, the message was introduced as a creative script from a digital campaign called The SmokeFree Initiative. The campaign was said to be created by either “a coalition of leading smoking cessation experts and creative writers” or “artificial intelligence.” The source information was prominently displayed in large, bolded fonts in an introductory paragraph on a separate screen prior to message presentation.

For the control message, ChatGPT was prompted to write a 500-word essay on how tobacco grows as a plant. Explicit instructions were given not to discuss the health effects of tobacco use. Only one version of this message was created, and its source was identified as FloraFacts, a botanical encyclopedia. The introduction and presentation of the control message followed the same procedure as in the smoking consequence message conditions.

All messages crafted by ChatGPT were carefully reviewed to ensure that they followed the prompts given and did not contain incorrect or inappropriate content. Editing was minimized to preserve the messages’ original quality for testing purposes. Sample messages are available in Appendix A.

### 2.5. Measures

#### 2.5.1. Smoking Status and Frequency

Participants reported whether they currently smoked at least 5 cigarettes a day, used to smoke at least 5 cigarettes a day for at least one year but did not currently smoke, or never smoked. This question was used by Prolific for screening and was asked again in the study to confirm eligibility. Those currently smoking also indicated the number of days smoking during the past 30 days and number of cigarettes smoked on an average day (5 or fewer, 6–10, 11–20, 21–30, and 31 or more).

#### 2.5.2. Dependence

Participants were asked how soon after waking up they would smoke their first cigarette. Answering options included within 5 min, 5 to 30 min, 31 to 60 min, and after 60 min. This question was a validated measure of nicotine dependence [40].

#### 2.5.3. Interest in Quitting

Participants indicated their level of interest in completely and permanently quitting smoking on a 5-point scale ranging from 1 not at all to 5 extremely. This measure was adopted from previous smoking cessation research [41,42].

#### 2.5.4. Thought Valence

Following established procedures [43], participants were instructed to briefly report the thoughts they had while reading the message. They were provided with eight textboxes and asked to put one thought in each box for as many thoughts as they could recall. For each of the thoughts listed, they were asked to indicate whether it was in favor of, against, or neutral/irrelevant to the message they just read. The positive and negative thoughts were tallied for each participant and an index of thought valence was constructed by taking the ratio of positive over negative thoughts. A constant of 1 was added to the number of negative thoughts to avoid an undefined denominator (*M* = 2.64, *SD* = 2.42).

#### 2.5.5. Emotional Responses

A measure adapted from existing research [30,44] asked participants how strongly they experienced different emotions while reading the message. Responses ranged from 1, not at all, to 5, a great deal. Three items measured fear responses, including fearful, afraid, and scared, and another three measured mirth, including happy, cheerful, and amused. Pertinent items were averaged into summary scores for fear (*M* = 2.06, *SD* = 1.22, α = 0.96) and mirth (*M* = 1.77, *SD* = 1.06, α = 0.87), respectively.

#### 2.5.6. Cognitive Responses

A validated 9-item scale [45] was adapted to measure perceived message effectiveness. Sample items included “The message is convincing” and “The message said something that is important to me.” Counterarguing was measured using a 4-item scale developed by Nabi and colleagues [46], with items such as “I found myself actively disagreeing with the message” and “I was looking for flaws in the message.” Another 4-item scale measured perceived threat to freedom [47], with items such as “The message threatened my freedom to choose” and “The message tried to manipulate me.” Responses were given on a 5-point Likert or Likert-type scale (e.g., 1 strongly agree to 5 strongly disagree). After proper recoding, the items for each scale were averaged into an overall score, where higher values indicated higher levels of the underlying construct (perceived message effectiveness, *M* = 3.36, *SD* = 0.84, α = 0.86; counterarguing, *M* = 2.27, *SD* = 0.89, α = 0.70; perceived threat to freedom, *M* = 2.18, *SD* = 1.07, α = 0.87).

#### 2.5.7. Risk Perceptions

On a scale from −5 much less likely to 5 much more likely, participants indicated the likelihood of personally experiencing the five health consequences of smoking featured in the messages as compared to nonsmokers their age and gender. These questions were constructed following examples for assessing behavioral outcome likelihood in the reasoned action literature [6,48]. Responses were averaged into a measure of targeted risk perceptions (*M* = 1.40, *SD* = 2.12, α = 0.91).

#### 2.5.8. Intentions to Quit

Consistent with measurement norms in the reasoned action approach [6,48], intentions to quit were measured with two questions asking about the likelihood of trying to quit and quitting completely and permanently in the next 6 months. Response options ranged from 1 extremely unlikely to 7 extremely likely and were averaged into an overall intention score (*M* = 4.70, *SD* = 1.73, *r* = 0.77, α = 0.87).

#### 2.5.9. Source Credibility

A measure developed by McCroskey and Teven [49] was used to assess source credibility. Six 7-point semantic differential items tapped into expertise (e.g., informed–uninformed), trustworthiness (e.g., honest–dishonest), and goodwill (e.g., cares about me–does not care about me). After proper recoding, the six items were averaged into a summary score where high values indicated higher credibility (*M* = 5.67, *SD* = 1.23, α = 0.91).

### 2.6. Analysis Plan

Descriptive analyses were conducted to assess sample characteristics. One-way analyses of variance (ANOVAs) and chi-square tests were conducted on smoking and demographic background variables to evaluate the success of randomization. Multivariate general linear models (GLMs) were estimated to examine condition differences in the outcome measures. One set of models included all five conditions to enable comparison against the control. A second set of models included only the four smoking cessation message conditions to test the main effects of, and potential interaction between, appeal type (threat vs. humor) and source attribution (AI vs. human experts). All models controlled for the number of days smoking in the past 30 days, the number of cigarettes smoked per day, and the time to first cigarette after waking to improve precision and reduce bias in estimation, as these variables can affect both information processing and risk perceptions. Analysis was performed using SPSS v.29 (IBM, Armonk, NY, USA). Statistical significance was determined at α < 0.05.

## 3. Results

A total of 550 participants screened into and completed the study. However, responses to the smoking status question revealed 52 former smokers, 11 never smokers, and 7 individuals who did not indicate their smoking status. These ineligible cases were removed from the sample. Characteristics of the final sample (*N* = 480) are summarized in Table 1. Of note, most of the participants were daily smokers (69%) who were at least moderately interested in quitting smoking (76%). Randomization checks revealed no significant difference in sample characteristics across the five study conditions (details not reported).

**Figure 1:**
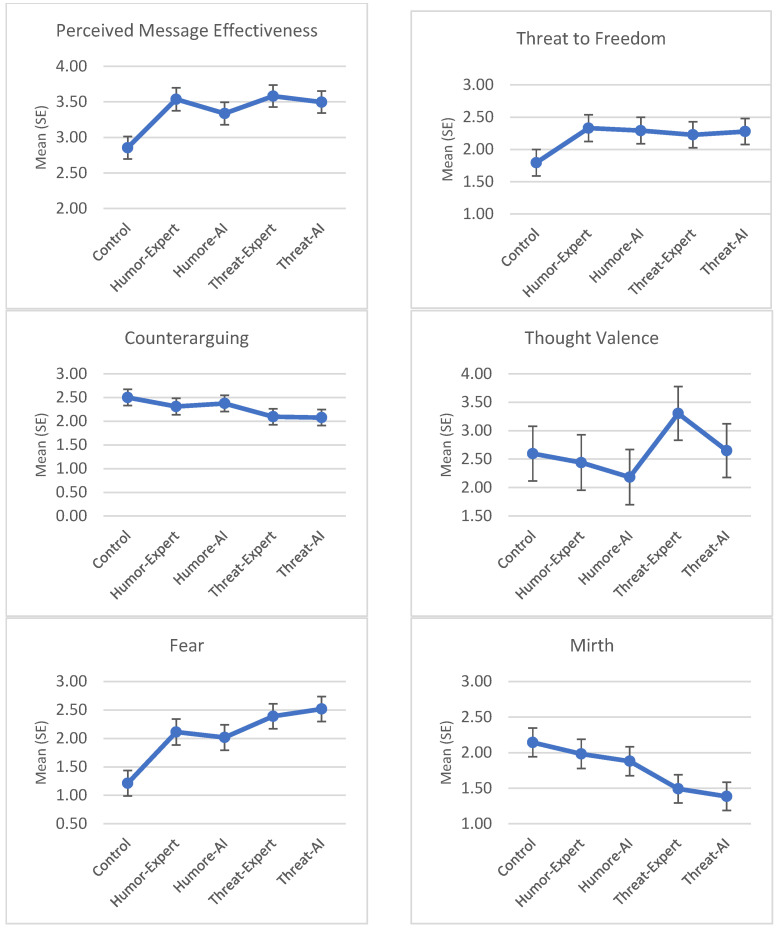

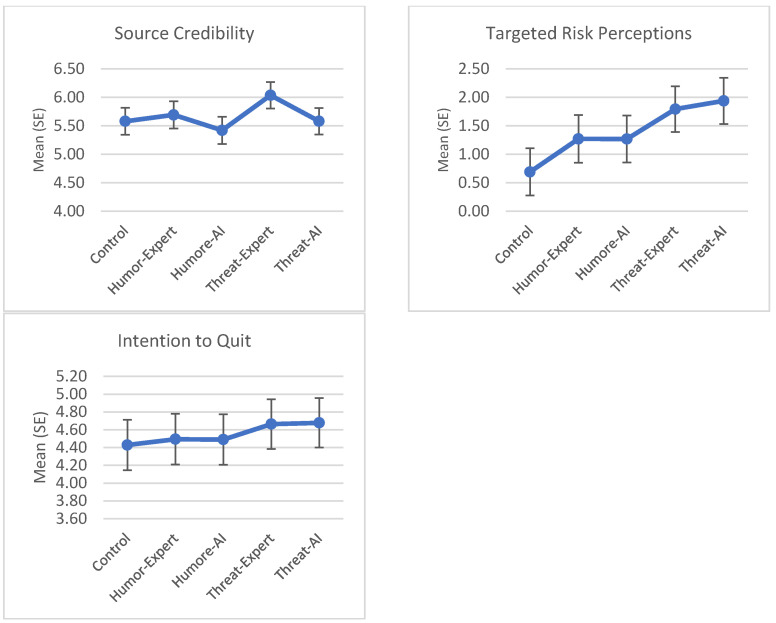
Condition means and standard errors in message response and quitting outcomes.

RQ1 asked whether the smoking cessation messages would elicit different cognitive and emotional responses than the control messages. Outcomes considered included perceived message effectiveness, thought valence, counterarguing, threat to freedom, fear, mirth, and perceived source credibility. Multivariate GLM with all five study conditions revealed a significant main effect of condition, *F* (28, 1876) = 6.541, *p* < 0.001, η_p_^2^ = 0.089. On the univariate level, a significant main effect of condition was observed with all seven outcomes. Details of test statistics are summarized in Table 2. Condition means and 95% confidence intervals are plotted in Figure 1. Pairwise comparisons showed that all four smoking cessation messages scored significantly higher than the control message on perceived message effectiveness, threat to freedom, and fear. The two threat messages scored significantly lower on counterarguing than the control message. The two humor messages scored significantly higher on mirth than the control message. Among the four smoking cessation messages, the threat-AI message produced significantly stronger fear than the humor-AI message; the humor-expert messages produced significantly higher mirth than the two threat messages; the humor-AI message produced significantly higher mirth than the threat-AI message; the threat-expert message produced significantly more positive thought valence than the humor-AI message; and the threat-expert message produced significantly higher perceived source credibility than the humor-AI message. No other pairwise comparisons were significant.

RQ2 asked whether cognitive and emotional responses to the smoking cessation messages would vary by message appeal and source manipulations. Multivariate GLM with only the four smoking cessation message conditions revealed a multivariate main effect of appeal type (humor vs. threat), *F* (7, 377) = 7.061, *p* < 0.001, η_p_^2^ = 0.117. The main effect of source identity (expert vs. AI) approached significance, *F* (7, 377) = 2.003, *p* = 0.054, η_p_^2^ = 0.036. The interaction between appeal type and source identity was not significant, *F* (7, 377) = 0.711, *p* = 0.663, η_p_^2^ = 0.013. Univariate test results are summarized in Table 2. Humor messages generated more counterarguing (*M* = 2.36 vs. 2.09), less fear (*M* = 2.049 vs. 2.446), greater mirth (*M* = 1.936 vs. 1.440), and less positive thought valence (*M* = 2.281 vs. 2.981) than the threat messages. An expert source led to greater perceived source credibility (*M* = 5.851 vs. 5.491) than an AI source. No other effects were significant.

RQ3 asked whether the smoking cessation messages would result in greater perceived risk with respect to the targeted health effects and intentions to quit smoking than the control message. Multivariate GLM with all five study conditions revealed a significant main effect of condition, *F* (8, 944) = 2.798, *p* = 0.005, η_p_^2^ = 0.023. On the univariate level, a significant main effect of condition was observed for targeted risk perceptions, but not for intentions to quit (see Table 2, Figure 1 for details). Pairwise comparisons showed that the threat messages generated significantly higher targeted risk perceptions than the control message. No other pairwise effects were significant.

RQ4 asked whether message appeal and source attribution would independently or jointly influence targeted risk perceptions and intentions to quit. In analysis with only the four smoking cessation message conditions, a multivariate main effect was observed for appeal type (humor vs. threat), *F* (2, 377) = 3.960, *p* = 0.020, η_p_^2^ = 0.021; but not for source attribution (AI vs. human experts), *F* (2, 377) = 0.054, *p* = 0.947, η_p_^2^ < 0.001. The interaction between appeal type and source attribution was also not significant, *F* (2, 377) = 0.064, *p* = 0.938, η_p_^2^ < 0.001. Univariate test results are presented in Table 2. Threat messages produced greater risk perceptions on the targeted health effects (*M* = 1.849 vs. 1.255) than humor messages. No other effects were significant.

## 4. Discussion

This study builds upon existing research to examine the use of AI-generated persuasive messages for smoking cessation campaigns. It contributes to the literature on AI communication on several fronts. First, it is one of the first studies to demonstrate that ChatGPT can generate relatively effective messages for smoking cessation campaigns. The AI-generated messages successfully elicited the targeted emotions and communicated health risks in a way that resonated with the audience. These messages significantly increased participants’ risk perceptions, underscoring their potential value for real-world campaigns. Previous research on AI-generated health messages has primarily focused on chatbots or AI assistants in one-on-one interactions [50], typically producing short messages [12,14]. This study expands on that approach, showing that AI can be used to develop campaign messages featuring specific content and appeals, with minimal human effort and cost, to effectively engage the target population and support public health causes.

Second, the AI-generated messages in this study continued the tradition of using threat and humor to communicate tobacco risks in campaign messaging [17,29,31]. Relative to a neutral, informational message about tobacco plant growth, both types of emotional appeal messages elicited greater perceived message effectiveness, threat to freedom, and fear. The humor and threat appeal messages also produced notably different outcomes. Threat messages produced greater fear but also more positive thought valence and greater perceived susceptibility to health risks. Humorous messages, on the other hand, produced greater mirth. These findings are consistent with past research that highlights threat messaging as a particularly effective strategy for tobacco risk communication [31,51]. Interestingly, threat messages in this study produced less counterarguing than humor messages. This finding runs counter to prior evidence that humor can reduce defensive responses [33,46]. One possibility is that the health effects featured in the messages in this study were all relatively novel, which made content-based counterarguing difficult, while the humorous execution might trigger resistance if the light touch was perceived as inappropriate. Overall, the general pattern of findings largely aligns with past research on smoking cessation messages, suggesting that AI-generated emotional messages can function similarly to those created by human message designers.

Third, this study contributed evidence on the effect of source attribution in the smoking cessation context. Overall, human experts were rated as more credible than AI, but source type made no difference in persuasive outcomes. Previous research often found that messages labeled as human-generated are preferred and seen as more persuasive [13,26]. However, our findings paint a different picture, showing no significant difference in perceived effectiveness between messages attributed to AI vs. human sources. Lim & Schmälzle [14] noted that mandatory AI source disclosure as a regulatory measure is still in the early stages of deliberation, and there is ongoing, broader debate about acceptable uses of AI in content creation. Our findings offer some assurance that revealing content as AI-generated may not lead to less favorable message reception.

In our study, smoking cessation messages enhanced smoking risk perceptions but did not significantly increase participants’ intentions to quit smoking. This is in some ways consistent with findings from Huang and Wang [10], which showed AI to be as effective as humans in influencing perceptions but less effective than humans in shaping behavioral intentions. This disconnect between perceptions and intentions may have complicated reasons in the broader AI–human comparison [10]. But from a behavioral prediction point of view, it may be that other factors, such as strong pro-smoking social norms, low perceived self-efficacy, and/or relevant individual characteristics somehow weakened the ability of message-induced risk perceptions to impact quitting intentions [6,52]. Future studies may explore AI-generated messages specifically targeting these other factors to examine their influence on intentions to quit smoking.

This study focused specifically on how humorous vs. threat messages attributed to AI vs. human sources could affect message responses, perceived risk, and intentions to quit smoking among individuals who currently smoke. Future research could expand this line of inquiry by examining AI’s ability to generate other types of strategically framed messages and emotional appeals both in the context of tobacco education campaigns and beyond.

The limitations of the study should be noted. First, the study mimics a typical message pretesting study, providing participants with a single exposure to a single test message. Such limited exposure might not be enough to generate meaningful changes in participants’ behavioral decision-making. This might be part of the reason why we did not observe any effect on quitting intentions. Second, the study was conducted with only textual messages. While this modality was adequate for our study purposes, it did not capture the full range of creative diversity in actual smoking cessation campaigns. Third, the study was a one-shot experiment with no behavioral follow-up. Whether behavioral effects could emerge over time remains uncertain. Finally, the study used a convenience online sample in the United States. Its representativeness of the U.S. adult smoking population is uncertain, particularly with respect to the minority and lower-education groups.

## 5. Conclusions

This study provides novel insights into the potential of AI-generated messaging in smoking cessation efforts. Our findings demonstrate that AI can create persuasive, relatively sophisticated campaign messages that resonate with audiences through both emotional and cognitive pathways. Moreover, the effectiveness of these messages did not significantly differ between AI and human sources, alleviating concern over the potential detrimental effects of AI source disclosure. Overall, our findings underscore AI’s potential utility as a message development tool in smoking cessation campaigns and highlight the need for further research into its application in other domains of health communication.

## Figures and Tables

**Table 1:** Sample Characteristics (*N* = 480).

		*n* (%)
Gender		
	Male	255 (53.1%)
	Female	221 (46%)
	Non-binary/third gender	4 (0.8%)
Race/Ethnicity		
	Non-Hispanic White	330 (68.8%)
	Non-Hispanic Black	74 (15.4%)
	Hispanic	50 (10.4%)
	Other race/ethnicity	26 (5.4%)
Age		
	18–34	107 (22.3%)
	35–44	139 (29%)
	45–54	134 (27.9%)
	55 or older	100 (20.8%)
Education		
	High school or less	73 (15.2%)
	Some college	173 (36%)
	Bachelor’s degree	184 (38.3%)
	Graduate degree	49 (10.2%)
	Prefer not to say	1 (0.2%)
Days smoking in past 30 days		
	0–15	63 (13.1%)
	16–29	86 (17.9%)
	30	331 (69%)
Cigarettes smoked per day		
	5 or fewer	115 (24%)
	6–10	158 (32.9%)
	11–20	150 (31.3%)
	21 or more	57 (11.9%)
Time to first cigarette after waking		
	Within 5 min	111 (23.1%)
	5–30 min	225 (46.9%)
	31–60 min	73 (15.2%)
	After 60 min	71 (14.8%)
Interest in quitting		
	Not at all	34 (7.1%)
	Slightly	81 (16.9%)
	A moderate amount	141 (29.4%)
	Very	96 (20%)
	Extremely	128 (26.7%)

**Table 2:** Tests of condition differences in message response and quitting outcomes.

	Model 1 (All Five Conditions)	Model 2 (Appeal X Source—Control Excluded)		
DV	Condition	Humor vs. Threat	Expert vs. Artificial Intelligence (AI)	Interaction
Perceived message effectiveness	*F* (4, 472) = 13.64, *p* < 0.001, η_p_^2^ = 0.104	*F* (1, 378) = 1.83, *p* = 0.177, η_p_^2^ = 0.005	*F* (1, 378) = 2.98, *p* = 0.085, η_p_^2^ = 0.008	*F* (1, 378) = 0.456, *p* = 0.500, η_p_^2^ = 0.001
Counterarguing	*F* (4, 472) = 4.44, *p* = 0.002, η_p_^2^ = 0.036	*F* (1, 378) = 9.15, *p* = 0.003, η_p_^2^ = 0.024	*F* (1, 378) = 0.040, *p* = 0.841, η_p_^2^ < 0.001	*F* (1, 378) = 0.166, *p* = 0.684, η_p_^2^ < 0.001
Threat to freedom	*F* (4, 472) = 4.44, *p* = 0.002, η_p_^2^ = 0.036	*F* (1, 378) = 0.523, *p* = 0.470, η_p_^2^ = 0.001	*F* (1, 378) = 001, *p* = 0.969, η_p_^2^ < 0.001	*F* (1, 378) = 0.204, *p* = 0.652, η_p_^2^ = 0.001
Thought valence	*F* (4, 472) = 2.91, *p* = 0.021, η_p_^2^ = 0.024	*F* (1, 378) = 8.81, *p* = 0.003, η_p_^2^ = 0.023	*F* (1, 378) = 3.47, *p* = 0.063, η_p_^2^ = 0.009	*F* (1, 378) = 0.837, *p* = 0.361, η_p_^2^ = 0.002
Fear	*F* (4, 472) = 20.06, *p* < 0.001, η_p_^2^ = 0.145	*F* (1, 378) = 10.32, *p* = 0.001, η_p_^2^ = 0.027	*F* (1, 378) = 0.026, *p* = 0.871, η_p_^2^ < 0.001	*F* (1, 378) = 0.758, *p* = 0.384, η_p_^2^ = 0.002
Mirth	*F* (4, 472) = 9.94, *p* < 0.001, η_p_^2^ = 0.078	*F* (1, 378) = 25.10, *p* < 0.001, η_p_^2^ = 0.062	*F* (1, 378) = 1.19, *p* = 0.277, η_p_^2^ = 0.003	*F* (1, 378) = 0.000, *p* = 0.989, η_p_^2^ < 0.001
Source credibility	*F* (4, 472) = 3.72, *p* = 0.005, η_p_^2^ = 0.031	*F* (1, 378) = 4.42, *p* = 0.036, η_p_^2^ = 0.012	*F* (1, 378) = 8.376, *p* = 0.004, η_p_^2^ = 0.022	*F* (1, 378) = 0.631, *p* = 0.427, η_p_^2^ = 0.002
Targeted risk perceptions	*F* (4, 472) = 3.72, *p* < 0.001, η_p_^2^ = 0.045	*F* (1, 378) = 7.48, *p* = 0.007, η_p_^2^ = 0.019	*F* (1, 378) = 0.107, *p* = 0.743, η_p_^2^ < 0.001	*F* (1, 378) = 0.128, *p* = 0.721, η_p_^2^ < 0.001
Intentions to quit	*F* (4, 472) = 0.609, *p* = 0.657, η_p_^2^ = 0.005	*F* (1, 378) = 1.47, *p* = 0.226, η_p_^2^ = 0.004	*F* (1, 378) = 0.002, *p* = 0.969, η_p_^2^ < 0.001	*F* (1, 378) = 0.008, *p* = 0.928, η_p_^2^ < 0.001

## Data Availability

Data and supplemental resources are available upon reasonable request to the corresponding author.

## Abbreviations

The following abbreviations are used in this manuscript:

AI	Artificial Intelligence
ANOVA	analyses of variance
GLM	General Linear Models

## Appendix A. Sample Messages

### Appendix A.1. Threat Message (1 of 3)

**The Urgency to Quit Smoking: Unveiling Hidden Health Consequences**

Smoking, once glamorized and socially accepted, now reveals a dark reality that cannot be ignored. Beyond the well-known risks to the lungs and heart, smoking exacts a heavy toll on various aspects of our health. This essay aims to shed light on lesser-known consequences of smoking, urging smokers to reflect upon the grave implications their habit has on their future well-being. By examining five particular health risks associated with smoking, including blindness, erectile dysfunction, hip fractures, rheumatoid arthritis, and gum disease, it becomes evident that the time to quit smoking is now.

*Going Blind*

The harrowing truth is that smoking can irreversibly damage our vision. Age-related macular degeneration, a leading cause of blindness in adults over 65, finds an unfortunate ally in the toxic fumes of cigarettes. Studies have shown that smokers are significantly more prone to developing this debilitating condition. By quitting smoking, one takes a crucial step toward preserving their sight and maintaining a life of visual richness.

*Erectile Dysfunction*

Smoking strikes at the very core of male sexual function, insidiously impairing blood flow throughout the body, including to the penis. Consequently, the narrowing of blood vessels induced by tobacco poses a direct threat to the ability to achieve and sustain erections. However, hope remains for those who choose to quit smoking. Breaking free from this harmful addiction can restore vascular health, reigniting the fires of intimacy and enhancing the overall quality of one’s life.

*Hip Fractures*

The relentless assault on the body initiated by smoking extends even to our bones. Smokers experience a loss of bone density at an accelerated rate, rendering them more susceptible to fractures, particularly in vulnerable areas such as the hip. By severing ties with cigarettes, individuals can slow down this bone-thinning process, preserving their skeletal strength and ensuring a future free from the limitations of fractured bones.

*Rheumatoid Arthritis*

Rheumatoid arthritis, a chronic inflammatory disease, manifests as excruciating joint pain and deformity, primarily afflicting women. Shockingly, smoking serves as one of the key contributors to this debilitating condition. By choosing to quit smoking, one not only mitigates the risk of developing rheumatoid arthritis but also delays its onset, allowing for a longer period of pain-free mobility and the ability to relish life’s simple joys.

*Gum Disease*

A grim consequence of smoking lies within our mouths—periodontitis, a destructive gum infection that mercilessly erodes the bone supporting our teeth. With smoking being a significant factor in the development of this insidious disease, tooth loss becomes an unfortunate reality for many smokers. By relinquishing cigarettes, individuals actively safeguard their oral health, preserving their pearly whites and the confidence that accompanies a healthy smile.

The ramifications of smoking extend far beyond the commonly acknowledged risks. The truth is somber and unforgiving, exposing smokers to the perils of blindness, erectile dysfunction, hip fractures, rheumatoid arthritis, and gum disease. Awareness of these hidden health consequences serves as a clarion call for smokers to take decisive action. The choice to quit smoking is not merely an act of self-preservation; it is a proclamation of reclaiming one’s health and future. Let us embrace this opportunity, break free from the shackles of tobacco, and embark on a journey toward a life filled with vitality, joy, and the promise of a brighter tomorrow.

### Appendix A.2. Humorous Message (1 of 3)

**Butt Out and Save Your Bits: A Comedic Plea to Quit Smoking**

Hey there, smokers! Are you tired of smelling like an ashtray and having all your clothes mistaken for vintage thrift store finds? Well, hold on to your lighters because I’m about to drop some knowledge bombs that will make you want to quit smoking faster than a toupee in a hurricane. Trust me, the health consequences of smoking aren’t just the same old boring lung cancer and heart disease stuff you have heard a million times. Prepare to be astounded and, hopefully, motivated to kick that nasty habit to the curb.

*Going Blind*

Listen up, peepers! Did you know that smoking can lead to blindness? Yep, you read that right. Smoking increases your risk of age-related macular degeneration, the leading cause of blindness in adults over the age of 65. So, if you want to continue binge-watching your favorite TV shows without subtitles, it is time to stub out that smoke and save your sight!

*Erectile Dysfunction*

Gentlemen, gather round. Smoking not only makes you smell like an ashtray, but it can also make your love life go up in smoke. You see, tobacco narrows blood vessels all over your body, including the ones that supply blood to your prized possession. But don’t despair! Quitting smoking can make a big difference, and who knows, you might just become the superhero of the bedroom once again!

*Hip Fractures*

Ladies and gentlemen, let’s talk about bone density. Did you know that smokers lose bone density faster than non-smokers? Yep, it’s true. And you know what that means? You’re at a higher risk of breaking something important, like your hip, while attempting to bust a move on the dance floor. Quitting smoking not only saves your bones but also saves you from becoming a viral video sensation on YouTube. Dance like nobody’s watching, not like a brittle twig!

*Rheumatoid Arthritis*

Attention, ladies! Smoking isn’t just an excellent way to ruin your complexion and make you smell like a chimney; it can also lead to rheumatoid arthritis. This delightful little condition causes painful swelling in your hands and feet, resulting in bone loss and joint deformity. And guess what? Smoking is one of the culprits. So, if you want to keep your hands nimble for texting and your feet ready for some killer dance moves, it’s time to quit smoking and embrace a healthier lifestyle.

*Gum Disease*

Smokers, here’s a friendly reminder: smoking can cost you more than just your lungs. It can also cost you your pearly whites. Smoking contributes to gum disease, which can make your gums say, ‘Bye-bye, teeth!’ Periodontitis, the gum infection caused by smoking, destroys the bone supporting your teeth, leading to tooth loss. So, if you want to keep smiling with confidence and avoid scaring small children, it’s time to put that cigarette out for good.

Alright, smokers, it’s time to face the music—or rather, face the consequences of your smoking habit. From going blind to losing your teeth, smoking wreaks havoc on your body in more ways than one. But fear not, my friends! Quitting smoking is your golden ticket to a healthier, happier life. So, be a rebel without a cigarette, break up with those tobacco sticks, and embrace a smoke-free future. Trust me, your body will thank you, and you’ll have plenty of new material for your stand-up comedy routine as a former smoker. Good luck, and remember, quitting smoking is like extinguishing a little fire within you—except this time, it’s a fire you’ll be glad to see go out!
